# Caspase‐1 inhibition prevents neuronal death by targeting the canonical inflammasome pathway of pyroptosis in a murine model of cerebral ischemia

**DOI:** 10.1111/cns.13384

**Published:** 2020-04-28

**Authors:** Jia Li, Jia‐Huan Hao, Di Yao, Rong Li, Xue‐Fei Li, Zhi‐Yuan Yu, Xiang Luo, Xing‐Hua Liu, Ming‐Huan Wang, Wei Wang

**Affiliations:** ^1^ Department of Neurology Tongji Hospital Tongji Medical College Huazhong University of Science and Technology Wuhan China; ^2^ Trauma Center/Department of Emergency and Trauma Surgery Tongji Hospital Tongji Medical College Huazhong University of Science and Technology Wuhan China; ^3^ Key Laboratory of Neurological Diseases of Chinese Ministry of Education The School of Basic Medicine Tongji Medical College Huazhong University of Science and Technology Wuhan China

**Keywords:** cerebral ischemic infarcts, gasdermin D, neuroprotection, pyroptosis, Vx765

## Abstract

**Aims:**

The involvement of pyroptosis in ischemic stroke remains to be established. Therefore, we used the specific pyroptosis inhibitor Vx765 as an experimental intervention target in a murine model of stroke.

**Methods:**

A total of 564 C57BL/6 mice were subjected to photothrombotic procedures and treated via gavage with Vx765 at 1‐hour post‐ischemia. We subsequently assessed the expression of Gasdermin D (GSDMD), inflammasomes, caspase‐1, and interleukin‐1β (IL‐1β) using immunofluorescence (IF) and Western blot (WB) analyses. We also examined ultrastructural changes of cortical neurons with transmission electron microscopy (TEM) and measured infarct volumes dynamically by magnetic resonance imaging (MRI). Moreover, we evaluated the neurologic deficits by modified neurological severity scores, the rotarod test, and Treadscan.

**Results:**

Elevated expression of GSDMD and GSDMD p30, the pore‐forming subunit, was evident in the peri‐ischemic region on days one and three post‐ischemia. The neuronal plasma, nuclear, and mitochondrial membranes showed ultrastructural damage at day three post‐stroke. Elevated expression of inflammasomes, caspase‐1, and IL‐1β was also present on days one and three post‐injury. There were significant differences between Vx765‐treated and vehicle groups in mean infarct volumes (14.36 vs 21.52 mm^3^; 12.34 vs 18.56 mm^3^; 4.13 vs 10.06 mm^3^; *P* < .05 at day one, three, and seven post‐surgery, respectively). Mice treated with Vx765 showed better motor recovery as assessed by serial behavior tests and had better neuronal survival, which was attributable to pyroptosis inhibition, as illustrated by downregulated expression of the effector protein GSDMD, inflammasomes, caspase‐1, and IL‐1β. Besides, treatment with Vx765 preserved neuronal membrane structures after the ischemic injury.

**Conclusions:**

Pyroptosis emerges as an important pathway for neuronal death in an acute ischemic stroke. Vx765, a low molecular weight drug that has proven safe in clinical epilepsy trials, has potential therapeutic value for cerebral ischemia by targeting the canonical inflammasome pathway of pyroptosis.

## 1 INTRODUCTION

Stroke is a lamentably common disease with a high mortality rate, and frequently resulting in severe and persistent disability in survivors. Despite decades of intensive efforts in searching for effective pharmacological treatments for ischemic stroke, very few such methods are currently considered effective in clinic.^1^ Obtaining a better understanding of the pathways for ischemic cell death in stroke remains a major challenge in modern neurobiology.

Pyroptosis, as distinct from the better‐known apoptosis, is a pathway of cell death that depends on the activation of caspase‐1, which was first observed in macrophages infected with *Shigella flexneri,* an intracellular bacteria.^2^ Two pyroptosis pathways are recognized based on their involved caspases enzymes, that is, the canonical (caspase‐1) and non‐canonical (caspase‐11) pathways. The canonical pathway is initiated by the activation of inflammasomes, which, in turn, activate caspase‐1, ultimately resulting in lytic cell death mediated by the effector protein Gasdermin D (GSDMD).^3^ This protein is constitutively expressed in an inactive state due to the autoinhibition of its C‐domain.^4^ Upon activation, the mobilized N‐domain of GSDMD forms membrane pores, which mediate the release of cytokines, cellular swelling due to water influx, membrane rupture, and eventually the release of all cellular contents, that is, lysis.^5^, ^6^, ^7^ Accumulating evidence points to a potentially important role of pyroptosis in the pathology of ischemic stroke. For example, clinical specimens of ischemic brain tissues showed elevated expression of inflammasomes.^8^ Furthermore, pharmacological inhibition of the pro‐inflammatory cytokine interleukin‐1β (IL‐1β) attenuated stroke severity in mice.^9^, ^10^ These results point to an important role of pyroptosis in the ischemic brain injury, which merits further investigation.

Thus, the pyroptosis pathway represents a promising pharmacological target for treating ischemic stroke. Vx765, an orally absorbed prodrug, is rapidly metabolized in vivo by non‐specific esterases to an active metabolite, VRT‐043198, which is a selective inhibitor of caspase‐1.^11^ The therapeutic potential of Vx765 was illustrated not only by its inhibition of IL‐1β releases from human monocytes in vitro, but also by its effect in reducing the serum levels of IL‐1β and other clinical biomarkers in murine models of dermatitis, arthritis,^11^ and temporal lobe epilepsy.^12^ As a brain penetrating caspase‐1 inhibitor with low toxicity, Vx765 is currently in a phase 2b clinical trial for drug‐resistant partial epilepsy and has thus already proven safe in humans. In a study of a murine multiple sclerosis model, Vx765 treatment diminished the upregulated expression of GSDMD and deactivated inflammasomes.^13^ As such, Vx765 may possess therapeutic efficacy in an ischemic stroke model and potentially also in clinical translation.

Here, we asked whether the canonical inflammasome pathway of pyroptosis indeed plays a central role in the pathology of cerebral ischemia, and if so, whether that pathological process might be ameliorated by treatment with the caspase‐1 inhibitor prodrug, Vx765. We used a combination of transmission electron microscopy (TEM), immunofluorescence (IF), Western blotting (WB), and in vivo magnetic resonance imaging to test the hypothesis that pharmacological inhibition of the canonical inflammasome pathway of pyroptosis would protect against neuronal loss and motor deficits in the murine photothrombotic (PT) ischemic stroke model.

## 2 MATERIALS AND METHODS

### 2.1 Animals and experimental design

Male C57BL/6 wild type mice, weighing 20‐28 g and aged 8‐12 weeks (Hunan SJA Laboratory Animal Co. Ltd), were housed in an SPF environment with a 12‐hour light‐dark cycle and adequate food and clean water. All animal studies were conducted with the permission and under the supervision of the Institute of Animal Care Committee in Tongji Hospital of Tongji Medical College, Huazhong University of Science and Technology, China (Institutional Review Board ID: TJ‐20170803).

Before surgical procedures, mice were randomly assigned into six groups (PT1d, PT3d, PT7d or sham‐operated, Vx765‐treated, or vehicle‐treated) using the random number generator in Graphpad. Based on the results of the previous study,^14^ and from the mean and standard deviation of outcomes from our pilot studies, we calculated that five animals per group would yield 80% power at a significant level of <0.05 with a 2‐sided *t *test. In our preliminary study, we evaluated infarct volumes using hematoxylin and eosin (HE) staining in the cortex on day three after PT. Since the PT lesion resulted in cortical infarcts of consistent size while bringing low mortality, we used 5‐7 animals per group (depending on the outcome being tested). A total of 564 wild type male mice were included in the experiments, plus 100 mice used for the pilot studies. To avoid sex and age differences (which would reduce statistical power), we decided to use adult male mice exclusively. Extensive efforts were made to minimize animals' discomfort, that is, adjusting isoflurane dose to reach the appropriate anesthesia level, applying a heating pad to promote recovery from anesthesia, and using flexible gavage tubes to avoid esophageal injuries from repeated intragastric drug administrations.

### 2.2 A mouse model of photothrombotic (PT)

Focal cerebral ischemia was induced following the standard PT protocol from JOVE.^15^ In brief, mice under isoflurane anesthesia received an intraperitoneal injection with Rose Bengal at a dose of 1 mg per 10 g body weight in subdued light. We freshly prepared the Rose Bengal solution at a concentration of 10 mg/mL. The anesthetized mice were then placed in a prone position in the stereotaxic frame (RWD Life Science Company). The target brain region, 2 mm lateral and 2 mm posterior to the bregma on the right hemisphere, was illuminated for 5 minutes with a cold light source (Schott, KL1500) starting 5 minutes after the Rose Bengal injection. The operated mice were subsequently placed on a heating pad until attaining full behavioral recovery and were then returned to their home cages.

### 2.3 Vx765 administration

Vx765 was fully dissolved in dimethyl sulfoxide to make stock solutions with a concentration of 500 mg/mL, which was stored at −80°C until use. After rewarming to room temperature, about 4 μL stock solution was mixed with sesame oil to a volume of 80 µL. The diluted solution was administered via gavage to each mouse at a dose of 100 mg/kg first at 1‐hour post‐surgery and again once a day until day seven. The dose of 100 mg/kg body weight was chosen according to a previous report literature^11^ in which mice received a single intragastric dose of 50, 100, and 200 mg/kg Vx765 before challenging with lipopolysaccharide. With decreased plasma IL‐1β levels as the endpoint, a plateau effect occurred at 100 mg/kg Vx765.

### 2.4 Immunofluorescence (IF) and hematoxylin and eosin staining (HE)

After euthanasia by intraperitoneal injection of pentobarbital overdose, mouse brains harvested and dehydrated by immersion in a 30% sucrose solution overnight at 4°C followed by cutting into 10 µm thick coronal sections using a cryostat. After blocking with donkey serum, sections were incubated overnight at 4°C, with primary antibodies followed by the application of fluorescent secondary antibodies for 1 hour at room temperature and mounting with antifade medium containing DAPI. The primary antibodies used in this study were as follows: GSDMD (1:100, mouse), NLRP1 (1:100, mouse), ASC (1:100, mouse), caspase‐1 (1:100, mouse), IL‐1β (1:100, hamster), NeuN (1:500, rabbit), GFAP (1:500, rabbit), NG2 (1:200, rabbit), Iba‐1 (1:500, goat), and Nlrp3 (1:200, mouse). Secondary antibodies (1:200) were Alexa Fluor@488 and Cy3 conjugated antibodies. Detailed information about antibodies is shown in Table S1.

A fluorescence microscope (Olympus, BX51) was used to capture images of the double‐labeled slices (with DAPI) for counting the immune‐positive cells. Images of the peri‐ischemic regions were taken as previously described.^16^ In brief, we regarded the areas extending about one mm beyond the infarct margins as peri‐ischemic regions, which contained signs of reactive gliosis (Figure S1). We selected 4‐6 fields in the peri‐ischemic region (around the infarct core) from 6 to 8 coronal slices of each mouse and captured microscopic images with a 40× objective lens. The.tiff file images of each group were blinded and analyzed manually by an experienced experimenter using Image J. Positive cells were defined as immune‐positive cells with nuclear staining to DAPI.

Z‐stack images of 0.5 µm thickness were captured from the 10 µm sections using a confocal microscope (Olympus, FV1200) equipped with a 100× oil immersion lens. Confocal setting parameters were kept the same during each IF immunofluorescence microscopy. The raw.oib file pictures were first processed with Image J, and the co‐labeling relationships noted in the orthogonal views.

The procedure of hematoxylin and eosin staining was as previously described.^17^ In brief, six to eight 10 μm slices were chosen with an inter‐slice interval of 500 µm for each of the five mice per group (vehicle vs treated) on day three. After serial staining of hematoxylin and eosin, these slices were photographed using a light microscope.

### 2.5 Western blots

The peri‐ischemic region from mice (sham vs PT, vehicle vs Vx765, n = 7 per group) was collected and extracted with ice‐cold RIPA lysis buffer. Portions containing 25 µg protein were loaded on the gel, processed for electrophoresis, and transferred to the NC membrane. The membranes were blocked with nonfat milk and incubated overnight at 4°C with the following primary antibodies, GSDMD (1:500, mouse), cleaved GSDMD (1:1000, rabbit), NLRP1 (1:500, mouse), NLRP3 (1:1000, mouse), NLRC4 (1:1000, rabbit), NLRP6 (1:1000, rabbit), ASC (1:1000, rabbit), caspase‐1 (1:500, mouse), IL‐1β (1:1000, rabbit), and HRP‐conjugated β‐actin (1:20 000). The HRP‐conjugated secondary antibodies were used at a 1:5000 dilution. Detailed information about the antibodies is shown in Table S1.

### 2.6 Transmission electron microscopy (TEM)

TEM was carried out following standard procedures. In brief, the peri‐infarct regions from the fresh brain samples were separated and quickly fixed by immersion in precooled 4% glutaraldehyde solution for 1 hour and then further trimmed to 1 mm^3^ tissue blocks for post‐fixation with glutaraldehyde and tannic acid. The tissue blocks were then embedded in neutral resin and cut with an ultramicrotome. After staining the ultrathin slices with lead acetate and uranium citrate, the slices were observed using an electron microscope (Hitachi, HT7700). Images were analyzed using Image J in an unbiased manner.

To TEM, the membranes of neurons normally appear as two dark lines separated by a narrow light‐colored gap. Among the intracellular organelles, we only analyzed the mitochondria, which were typically round‐, oval‐, or rod‐shaped and presented the double‐membrane structure with intima ridges. The density of neuronal mitochondria was calculated as the number of intracellular mitochondria divided by the intracellular cell area minus the nuclear area in the profile. We also counted synapses defined by the presence of post‐synaptic electron‐dense deposits as opposed to presynaptic vesicles.

### 2.7 Enzymatic colorimetric assays (ELISA)

The levels of IL‐1β and IL‐18 in the ischemic cortex were assessed using ELISA kits following the manufacturers' instructions. In brief, the cortex of the ipsilateral hemisphere was isolated and homogenized (100 mg tissue per mL PBS) using an electric homogenizer. After two freeze‐thaw cycles to break down the membranes, the supernatant was collected for analysis.

### 2.8 Magnetic resonance imaging

The vehicle and treated groups (five mice per group) underwent MRI scanning under isoflurane anesthesia on days one, three, and seven after surgery. The 7 Tesla Bruker AVANCE II Biospec MRI machine equipped with a surface coil specific for mouse brain scanning (Bruker Corp.) was applied. The image parameters for T2‐weighted images (T2WIs) were effective echo time/repetition time (eTE/TR) = 36/2000 ms, while the parameters of diffusion‐weighted images (DWIs) were TE/TR = 28/5000 ms using six different B values of 0, 200, 300, 500, 800, and 1000 s/mm^2^ in each slice direction. The field of view was 20 × 20 mm^2^ for 14 slices with a thickness of 0.8 mm. The resolution in dots per inch (DPI) was 256 × 256 of the final T2WIs and 128 × 128 for the DWIs.

The DICOM images were analyzed using the Image J software by an experimenter blinded to the treatment group. The infarct area in each T2WI and DWI slice was identified by its high signals. The infarct volumes were quantified as previously described.^14^ The lesion area of each slice was corrected for brain edema by multiplying the area ratio of the ipsilateral hemisphere vs the contralateral hemisphere. The total infarct volume was obtained from the summation of lesion areas multiplied by the 0.8 mm image slice thickness.

### 2.9 Behavioral assessments

We used the modified neurological severity scores (mNSS), rotarod tests, and gait analysis to evaluate the motor recovery as previously described.^18^, ^19^, ^20^ In particular, two groups (vehicle and treated, 12 mice per group) were trained for three consecutive days before the surgery and assessed daily from day one to seven post‐surgery. For the assessment of mNSS, mice were trained to cross the beam without hesitation. After suspending the mice by their tails, ipsilateral limb flexion exceeding 100 degrees relative to the antero‐caudal body axis was regarded as indicating a neurological deficit. In the placement test, we determined whether the mice could walk in a straight line. Finally, in the beam test, the presence of a motor deficit was indicated by the inability to pass across the beam smoothly. The total mNSS was the sum of the three binary items to a maximum total score of fourteen.

For rotarod tests, mice were trained to stay on the rotarod for at least 5 minutes after gradual acceleration from five revolutions per minute (rpm) to 40 rpm. Mice were evaluated three times at intervals of 15 minutes for adequate rest. The latency before falling from the rotarod results was calculated as the mean value of three trial results.

For gait analysis (Clever Sys., Treadscan), mice were trained in forced running on a treadmill against a brightly illuminated background. Videos were captured with a high‐speed camera in the ventral view for the analysis of strides. Each trial recording started when the mouse began to keep up with the speed of the treadmill. The data were later analyzed using the Treadscan software.

### 2.10 Statistics

All the outcome analyses were carried out by an independent investigator blinded to the groups. Data are expressed as mean ± SEM. IBM SPSS Statistics 22 or Prism 7.0 was used for statistical analysis. Kolmogorov‐Smirnov test was used to test whether the value comes from a Gaussian distribution and *P* > .05 was considered as passed the normality test. Then, significance was determined by ANOVA and Dunnett's test to compare differences between PT (days 1, 3, and 7) and the sham‐operated group. The difference between vehicle and treated groups was determined by Student's *t *test, or by one‐way ANOVA with repeated measures in the comparison of behavioral performance and MRI cortical lesions. Mann‐Whitney *U* test was used in the comparison of the intracellular mitochondrial area distribution (PT3d vs sham, vehicle vs sham) and mitochondrial density (vehicle vs treated group). *P* < .05 was considered statistically significant.

## 3 RESULTS

### 3.1 Involvement of neuronal pyroptosis in ischemic damage

**FIGURE 1:**
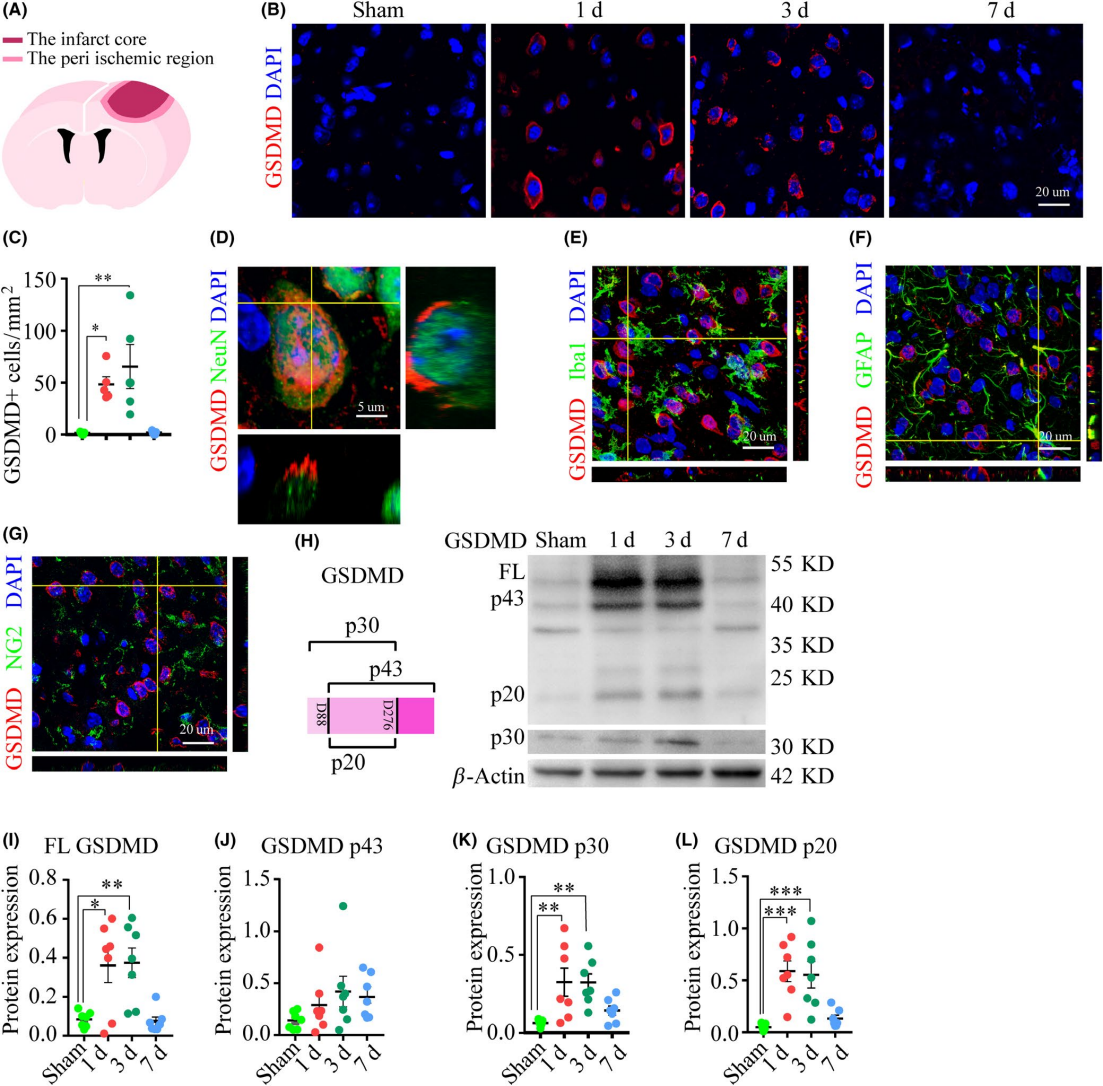
The involvement of pyroptotic cells in the peri‐ischemic regions. (A) A schematic graph depicting the cortical lesion. (B) Series of double‐stained images, GSDMD (red), DAPI (blue), in the peri‐ischemic region. (C) Time course of the number of GSDMD‐positive cells in the peri‐ischemic region, and data are the mean results from five mice per group, *P < .05, and **P < .01, compared with the sham‐operated group. (D‐G) Representative images showed the co‐labeling relationship of GSDMD/NeuN (neurons), GSDMD/Iba‐1 (microglia), GSDMD/GFAP (astrocytes), and GSDMD/NG2 (oligodendrocytes). (H‐L) WB images and analysis of full‐length GSDMD, as well as cleavage products P43, p30, and p20. Data are the mean results from seven mice, *P < .05, **P < .01, and ***P < .001, compared with the sham‐operated group

Several studies have reported that GSDMD is the determining factor in pyroptotic cell death.^3^, ^5^, ^21^ To assess the involvement of pyroptosis in ischemic brain damage, we quantified the expression of the pore‐forming subunit GSDMD on days one, three, and seven post‐ischemia by WB and IF, compared with the sham‐operated group. A sharp rise in GSDMD expression was observed via IF on day one, which was sustained on day three but had been normalized on day seven (Figure 1A‐C). We then explored the specified cell type that underwent pyroptosis. Our results showed that GSDMD was mainly co‐labeled in NeuN‐positive cells (neurons), rather than Iba1‐ (microglia), GFAP‐ (astrocytes), or NG2‐immunoreactive cells (oligodendrocytes) (Figure 1D‐G). So far, two cleavage sites have been identified in the processing pathway for murine GSDMD, that is, D276 and D88,^22^, ^23^ which give rise to three different proteolytic cleavage products, known as p43, p30, and p20. The expression of the full‐length GSDMD, as well as that of p30 and p20, was all dramatically increased on days one and three post‐PT (Figure 1H,I,K,L). All these results indicated that viable neurons in the peri‐ischemic region were undergoing pyroptosis during this acute post‐ischemia phase.

**FIGURE 2:**
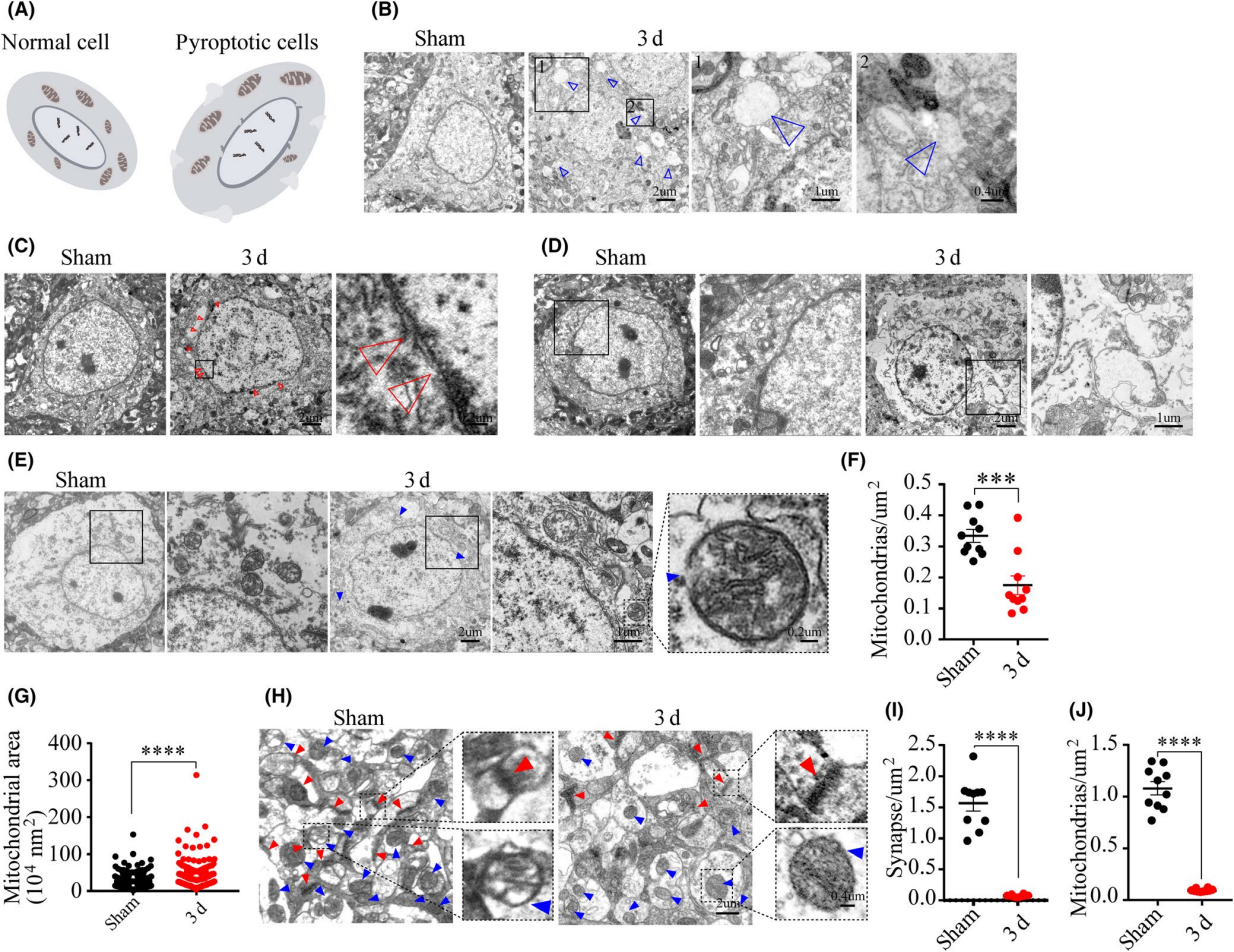
The ultrastructural damages of cells in the peri‐ischemic region. (A) A schematic graph shows the ultrastructural damage of neurons. (B) Representative images showed the difference between the plasma membrane of the PT group and sham‐operated group, and the hollow blue arrowheads indicate holes. (C‐D) Images depicted details in the nuclear membrane completeness and cellular contents, and the hollow red arrowheads point to the nuclear debris. (E‐G) Images and analysis show mitochondrial structural details inside neurons, n = 335 mitochondria from ten neurons in the sham group, n = 161 mitochondria from ten neurons in the ischemic group, and blue arrowheads pointed the damaged mitochondria. (H‐J) Images and analysis of the synaptic and mitochondrial density. Red arrowheads point to synapses and blue arrowheads point to mitochondria. ***P < .001 and ****P = .0001, compared with the sham‐operated group

The subcellular events mediated by activated GSDMD in pyroptotic macrophages reportedly consist of degradation of mitochondria and other membranous intracellular organelles that precede the lethal plasma membrane rupture.^24^ Therefore, we investigated the ultrastructural damages of neurons in the peri‐ischemic region using TEM, focusing on the integrity of the plasma, nuclear, and mitochondrial membranes (Figure 2A). Since the expression and processing of GSDMD peaked on day three, we made a comparison between post‐ischemia and sham‐operated groups on that day. We observed bubbles and large holes in the plasma membrane of neurons from the ischemic group, in contrast to the linear and intact plasma membrane from the sham group (Figure 2B). Moreover, the outer layer of the nuclear membrane was compromised by ischemic damage, as shown by its incompleteness and the presence of floating debris (Figure 2C). The rupture of the plasma membrane *in extremis* led to the extravasation of cellular contents, as shown by the reduced abundance of intracellular dense particles (Figure 2D). Some mitochondria in the neurons from the ischemic group appeared to be in certain respects normal, with a regular size, round shape, and intima crest, although the double‐layer membrane was completely destroyed (Figure 2E), with overall depletion and swelling of mitochondria (Figure 2F,G). There was also a decreased abundance of synapses in the peri‐ischemic region (Figure 2H‐J).

### 3.2 The ischemic signal triggered the canonical inflammasome pathway of pyroptosis

**FIGURE 3:**
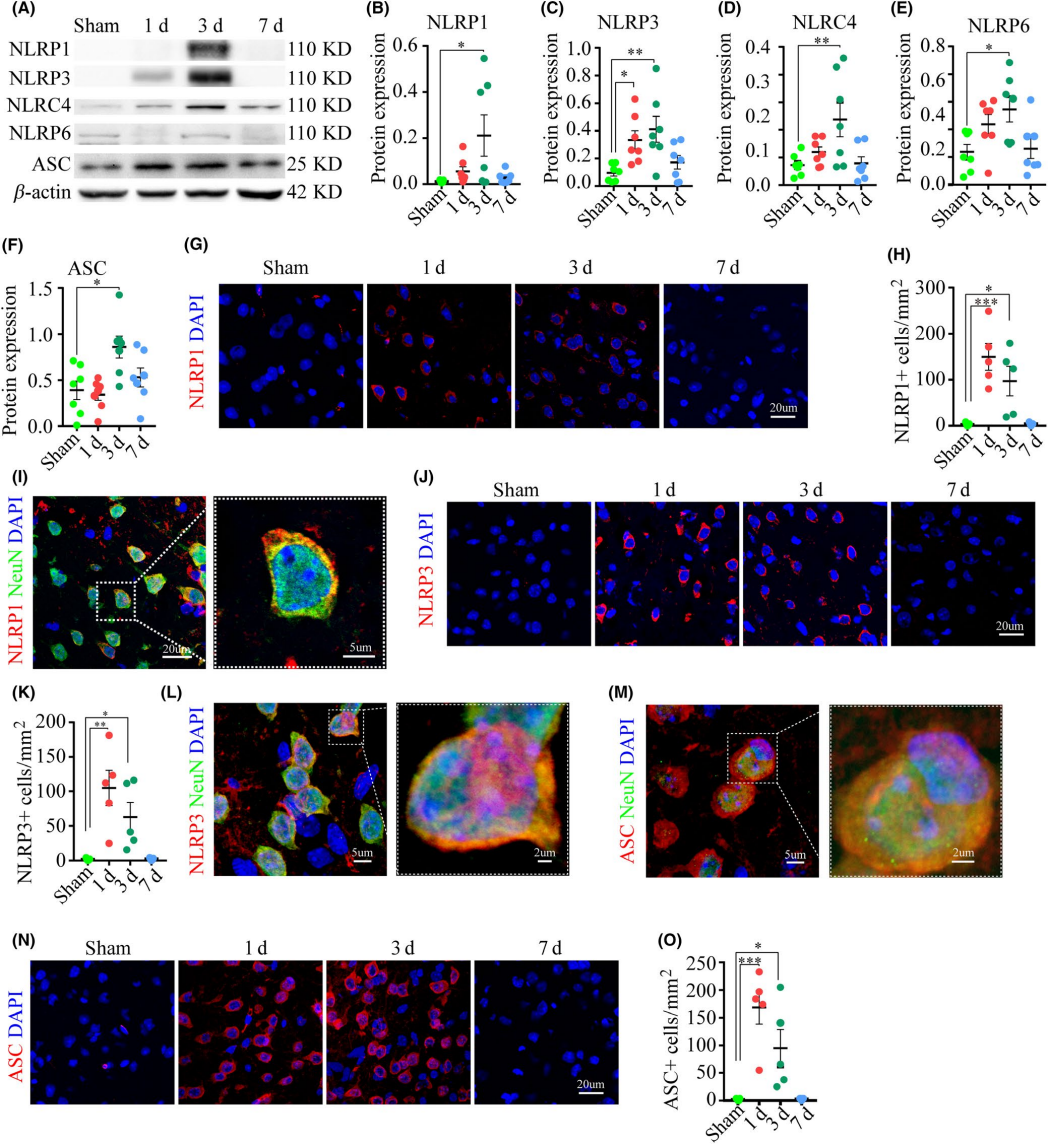
The activation of inflammasomes following the ischemic injury. (A‐F) Representative WB images and analysis displayed the expression of inflammasomes and the adaptor ASC, n = seven mice per group. (G‐H) Images and analysis demonstrated the number of NLRP1 IF‐positive cells. (I) Images displayed the co‐labeled NLRP1 and NeuN. (J‐O) Images and analyses displayed the expression of NLRP3 and ASC and their co‐labeling with NeuN. *P < .05, **P < .01, and ***P < .001, compared with the sham‐operated group

The inflammasomes are a group of proteins with a complex multiprotein structure, which serve to sense endogenous “danger” signals and mediate the process of pyroptosis.^25^, ^26^ We asked whether ischemic signals would induce canonical pyroptosis via the activation of inflammasomes. To test this, we used WB to measure the concentrations of inflammasomes from NLR family (NLRP1, NLRP3, NLRC4, and NLRP6). NLRP3 expression in the peri‐ischemic region rose sharply on day one and remained high on day three, when NLRP1, NLRP3, NLRC4, and NLRP6 expression was also high (Figure 3A‐E). Apoptosis‐associated speck‐like protein containing a CARD (ASC) serves as a signal amplification mechanism for NLRP3 and NLRC4.^27^ The peri‐ischemic expression of ASC was significantly increased on day three, which was consistent with the concomitantly increased expression of NLRP3 and NLRC4 (Figure 3A,F). There were increased numbers of cells positive for NLRP1‐, NLRP3‐, and ASC‐IF on days one and three in the peri‐ischemic region (Figure 3G,H,J,K,N,O). Moreover, NLRP1, NLRP3, and ASC proteins co‐labeled within NeuN‐positive cells (neurons) (Figure 3I,J,M).

**FIGURE 4:**
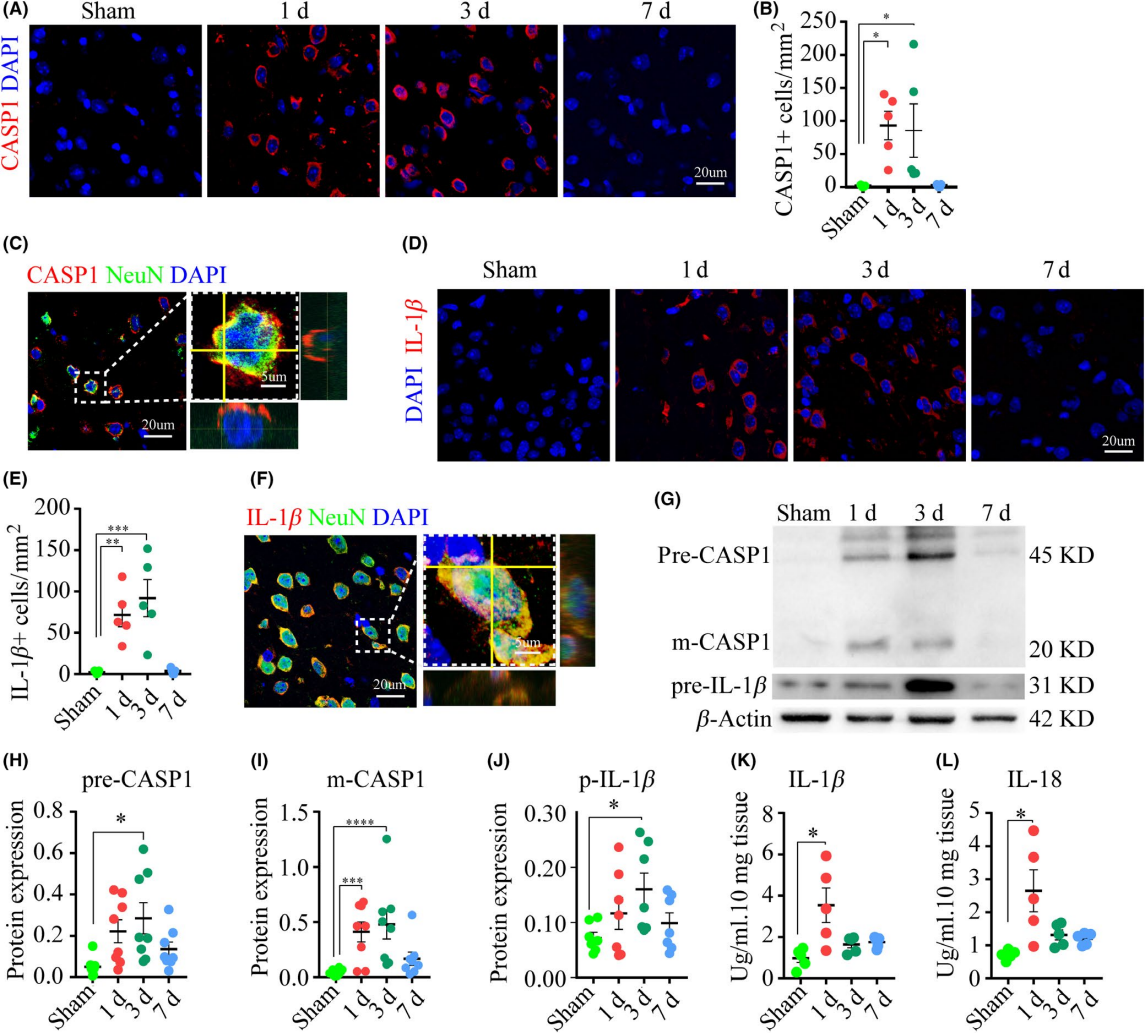
Pro‐inflammatory caspase and cytokines are activated following ischemia. (A‐B) Images and analysis depicted the expression of caspase‐1, n = five mice per group. (C) Images of double‐stained caspase‐1 and NeuN. (D‐E) Series of IF images displayed Il‐1β expression. (F) Images of double‐stained IL‐1β and NeuN. (G‐J) WB images and analysis showed the expression of caspase‐1 and IL‐1β. (K‐L) ELISA analysis of IL‐1β and IL‐18 in the ipsilateral cortex, n = five mice/group. *P < .05, **P < .01, ***P < .001, and ****P = .0001, compared with the sham‐operated group

The activation of inflammasomes mediates the maturation and activation of caspase‐1, the key mediator of canonical pyroptosis.^7^, ^28^ We found a sharp rise of caspase‐1 expression by IF on days one and three post‐injury, in parallel with the dynamic changes of inflammasomes (Figure 4A,B). Further analysis showed that caspase‐1 co‐labeled within NeuN‐positive cells (neurons) (Figure 4C). There was an elevated expression of the caspase‐1 precursor on day three and a sustained elevation of the cleavage product p20 on days one and three, which had both been normalized by day seven (Figure 4G,I). We also found that the dynamic expression of IL‐1β by IF was consistent with that of caspase‐1, with a sharp rise on day one that persisted to day three (Figure 4D,E). Likewise, the expression of the IL‐1β precursor was significantly elevated on day three post‐ischemia by WB (Figure 4G,J). ELISA results showed that there were increased levels of IL‐1β and IL‐18 in the ipsilateral cortex on day one (Figure 4K,L).

### 3.3 Pyroptosis specified inhibitor Vx765 attenuated ischemic damages caused by photothrombotic

**FIGURE 5:**
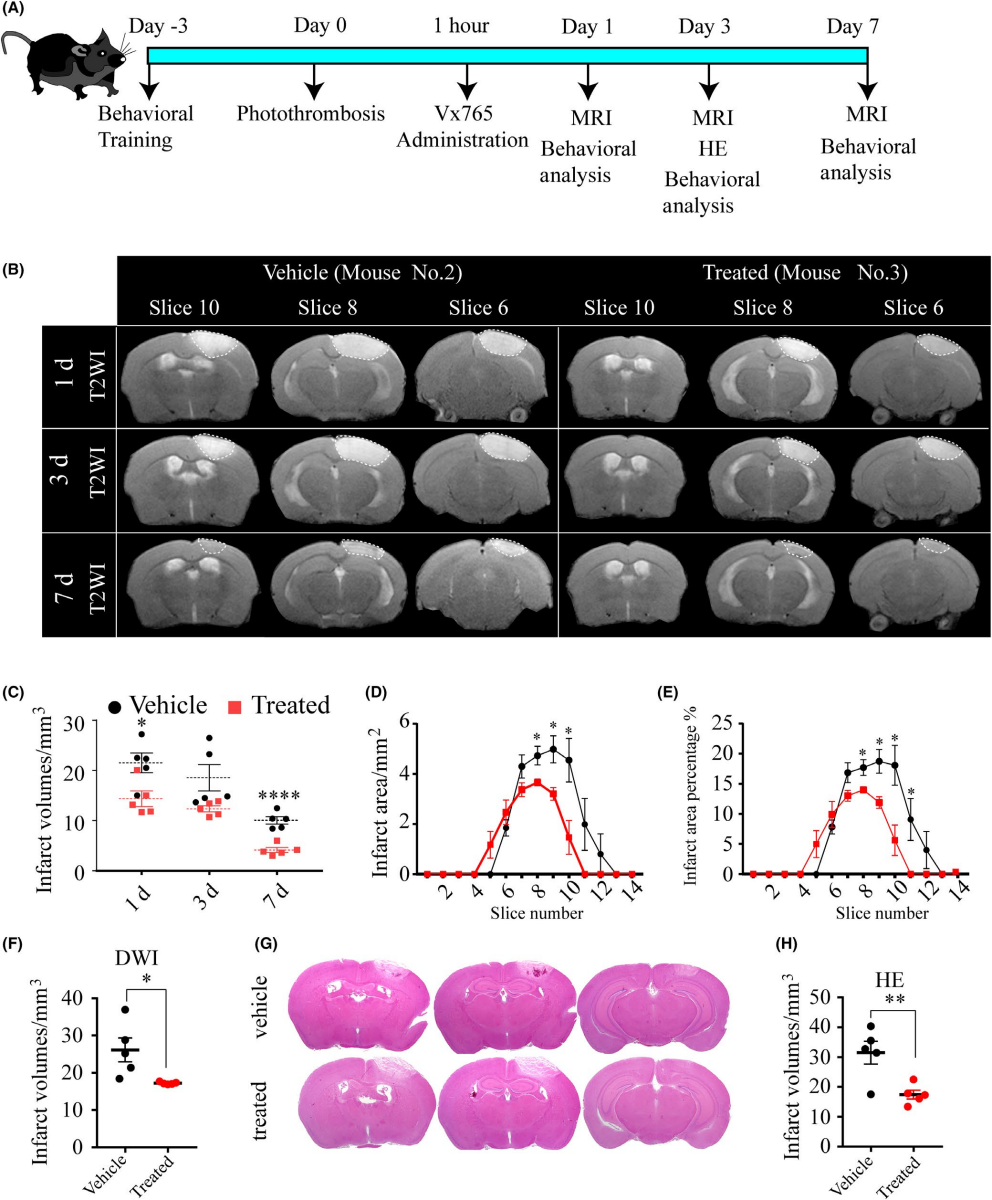
Selective caspase‐1 inhibitor Vx765 decreases infarct sizes. (A) The schematic diagram displayed the experimental design. (B) Representative T2WIs show the dynamic changes of the infarct areas of mouse No. 2 in the vehicle group and mouse No. 3 in the treated group. The dotted area of the images is intended to highlight the lesion. (C) The summed infarct volumes of each mice in the serial scanning on days one, three, and seven based on T2WIs, n = five mice per group. (D‐E) The summed infarct areas and infarct area percentages on day three based on T2WIs. (F) The analysis of infarct sizes on day three based on DWIs. (G‐H) HE stained images and analysis of infarct sizes on day three, n = five mice per group. *P < .05, **P < .01, and ****P = .0001, compared with the vehicle group

Vx765 is a specific pyroptosis inhibitor prodrug which demonstrated no protective activity in models of apoptosis.^11^ PT mice were treated for 1 week with Vx765 via gavage at a daily dose of 100 mg/kg beginning on 1‐hour post‐surgery. To reveal the salubrious effects of this treatment, we utilized 7 Tesla MRI and hematoxylin and eosin staining to measure the infarct sizes, behavioral assessment to detect neurological deficits, WB and IF examination to assess the expression of pyroptosis‐associated proteins, and TEM to observe the ultrastructural changes, in the comparison between vehicle‐ and Vx765‐treated groups (Figure 5A).

MRI results showed rather smaller cortical lesions in the treated group on days one, three, and seven, as shown by the smaller high signal area and fewer affected slices in the ipsilateral cortex on T2WIs, compared with the vehicle group (Figure 5B). We emphasized the lesion boundaries using dotted lines (Figure 5B). In the treated group, there was an approximately 33% relative reduction in infarct volume on days one (treated, 14.36 ± 1.55 mm^3^; vehicle, 21.52 ± 1.97 mm^3^; *P* = .021) and a 60% reduction on day seven (treated, 4.13 ± 0.50 mm^3^; vehicle, 10.06 ± 0.74 mm^3^; *P* = .0001) (Figure 5C). Further analysis of the T2WIs on day three revealed an overall smaller infarct area in the affected slices of the treated group, with or without the correction for the total slice area (Figure 5D,E). Analysis of infarct sizes on DWIs on day three supported this observation of decreased infarct sizes in the treated group (treated, 17.21 ± 0.15 mm^3^; vehicle, 26.14 ± 3.22 mm^3^; *P* = .024) (Figure 5F). Hematoxylin and eosin staining for assessment ex vivo of infarct sizes three days post‐injury likewise showed that Vx765‐treated PT mice had much smaller infarct sizes than did vehicle mice (Figure 5G,H).

**FIGURE 6:**
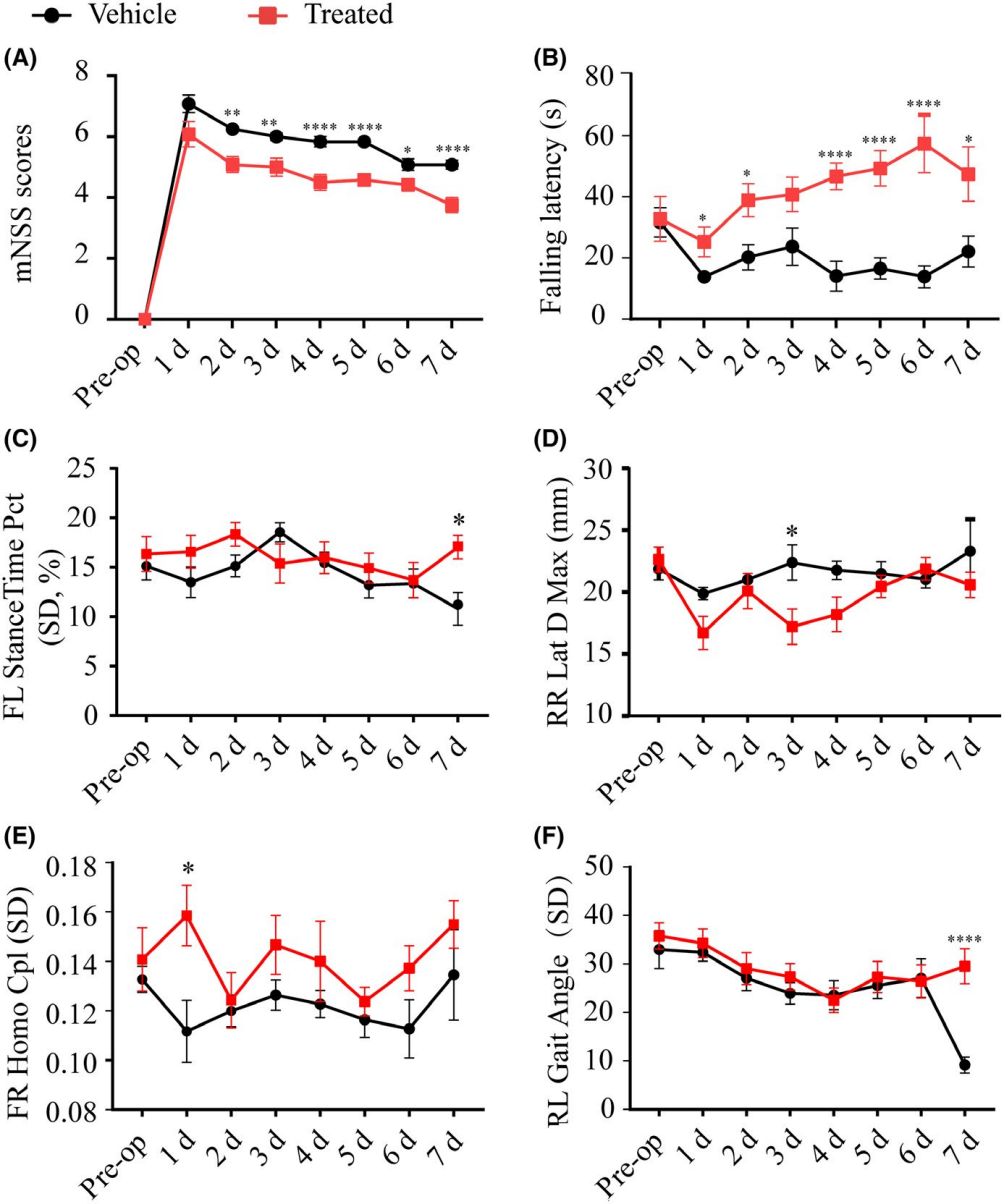
Vx765 administration ameliorated motor function after ischemia. (A) mNSS scores between the treated group and the vehicle group. (B) The latency to falling of the two groups observed in the rotarod test. (C‐F) Treadscan analysis of stance time percentage, lateral deviation, homologous CPL (coupling parameter), and gait angle, n = 12 mice per group. *P < .05, **P < .01, and ****P = .0001, compared with the vehicle group

We next asked whether the reduction in infarct sizes from Vx765 would be associated with attenuated neurological deficits in the PT model. The mNSS was significantly reduced by Vx765 treatment on days four, five, and seven (Figure 6A). Mice in the treated group managed to stay longer on the rotarod, compared with the mice from the vehicle group, beginning on day three (Figure 6B). Furthermore, the rotarod test showed that Vx765 treatment promoted motor learning behavior; the latency to falling increased with successive trials in the treated group, while declining in the vehicle group (Figure 6B). Treadscan analysis of subtle coordination showed that mice with drug treatment had a higher homologous coupling parameter on day one, decreased maximal lateral deviation on day three, and increased stance length and gait angle on day seven, all in comparison with the vehicle group (Figure 6C‐F).

### 3.4 Vx765 promoted neuronal survival by inhibiting pyroptosis

**FIGURE 7:**
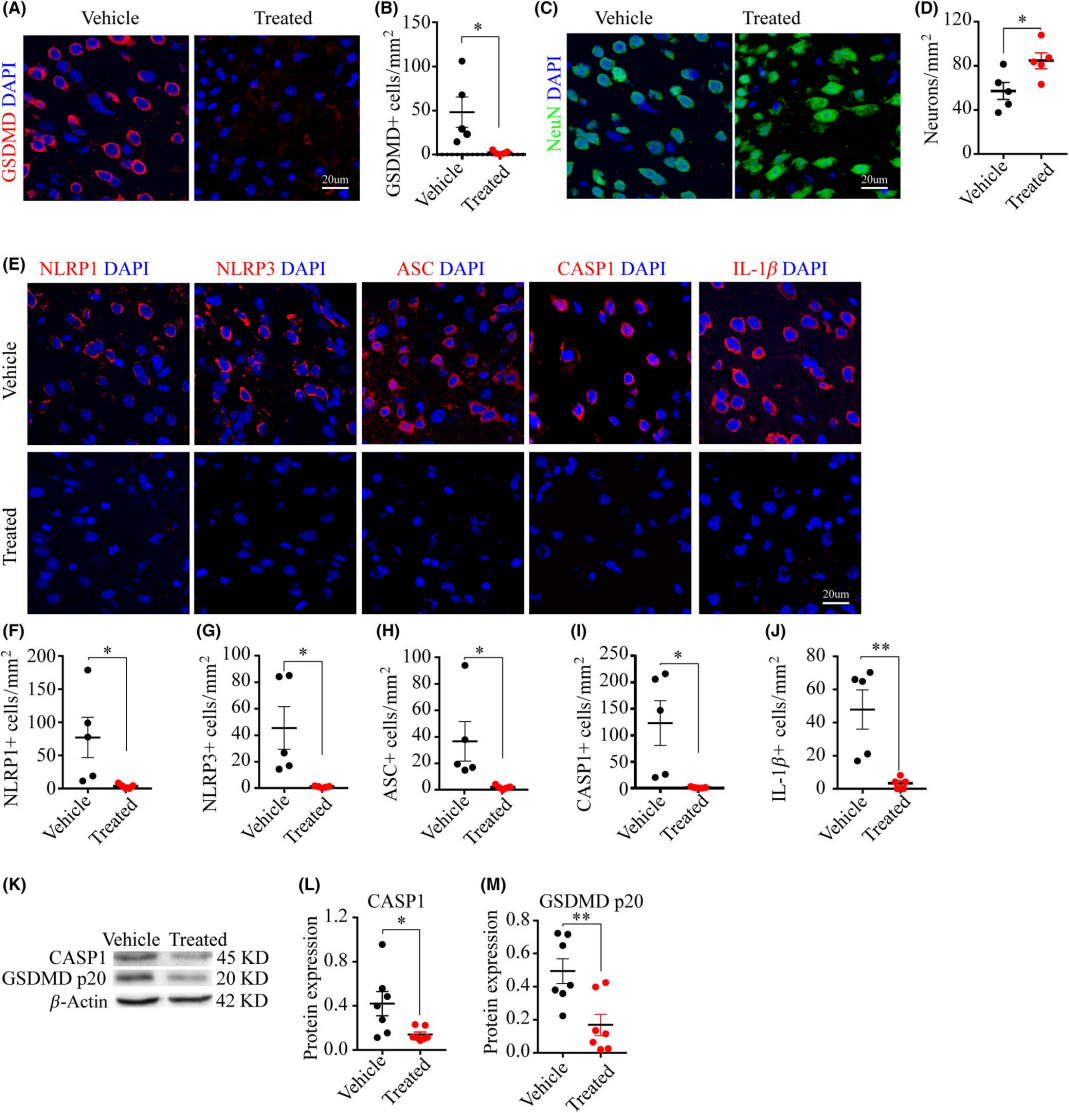
Vx765 promoted neuronal survival and inhibit the activation of Gsdmd as well as pyroptosis‐associated protein. (A‐D) Images and analysis demonstrated the number of GSDMD‐ and NeuN‐IF‐positive cells in the PIR at day three, n = five mice per group. (E‐J) Images and analyses showed the number of NLRP1, NLRP3, ASC, caspase‐1, and IL‐1β‐IF‐positive cells in Vx765 and vehicle‐treated group at day three post‐stroke, n = five mice per group. (K‐M) WB images and expression analyses showed the expression of precursor caspase‐1 and GSDMD p20 subunits in the Vx765 and vehicle‐treated group at day three post‐stroke, n = seven mice per group. *P < .05 and **P < .01, compared with the vehicle group

To assess the neuroprotective effect of Vx765, we first analyzed the protein expression of the effector GSDMD by IF and WB between vehicle and treated group at day three post‐injury, since there was an overtly upregulated expression of GSDMD at this time point. The increased expression of GSDMD in neurons was almost entirely suppressed by three days' treatment with Vx765 (Figure 7A,B). The treatment rescued the neuronal population in the peri‐ischemic region in the treated group (Figure 7C,D). Indeed, the treatment suppressed the canonical inflammasome of pyroptosis, inhibiting the expression of the inflammasomes NLRP1, NLRP3, the adaptor ASC, the pro‐inflammatory caspase‐1, and the pro‐inflammatory cytokine IL‐1β (Figure 7E‐J). To WB, the treatment decreased the expression of the caspase‐1 precursor and GSDMD p20 subunit (Figure 7K‐M,L). The protein expression of NLRP1, NLRP3, ASC, caspase‐1 p20, and IL‐1β is shown in Figure S2.

**FIGURE 8:**
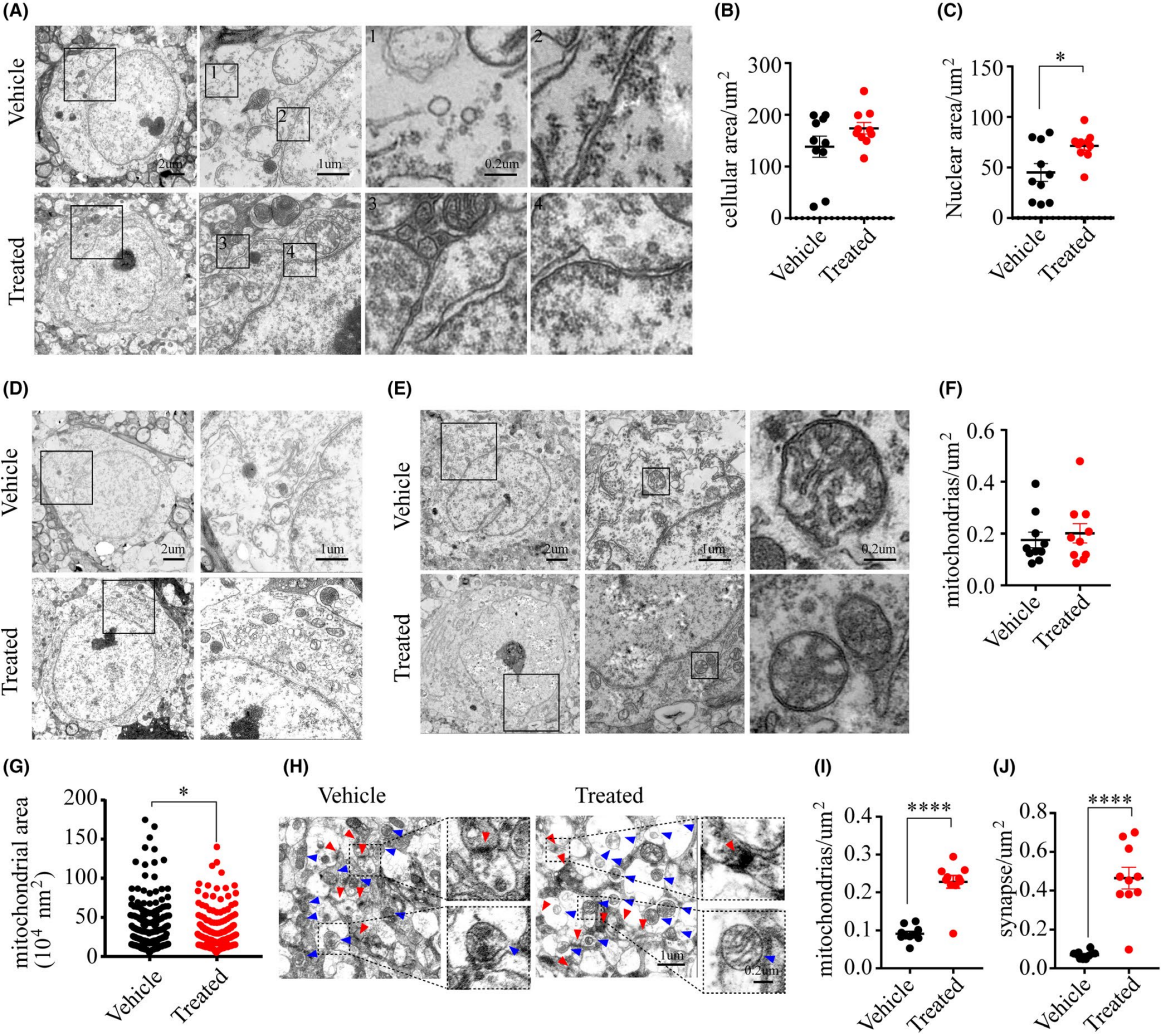
Vx765 treatment ameliorated ischemic ultrastructural damage. (A‐C) The plasma membrane and the nuclear membrane of neurons in the PIR in the treated group and the vehicle group on day three post‐injury. (D) Images showed cellular contents in neurons. (E‐G) Images and analysis showed mitochondrial morphology and number. (H‐J) Mitochondrial and synaptic density in the PIR. *P < .05 and ****P = .0001, compared with the vehicle group

Finally, our exploration of treatment effects on neuronal ultrastructures at day three post‐injury showed that peri‐ischemic neurons in the Vx765 mice had better preservation of plasma membranes and nuclear membranes, compared with the untreated mice (Figure 8A‐C). The drug treatment also preserved mitochondrial membrane structure and cellular contents (Figure 8D‐G). Moreover, the treatment protected against the synaptic loss (Figure 8H‐J).

## 4 DISCUSSION

The pyroptosis pathway has drawn increasing attention in studies of CNS injuries. For example, treatment with a pyroptosis inhibitor preserved axons in the spinal cord lesions of a murine multiple sclerosis model^29^ and ameliorated neurobehavioral performance in rodent models of neurodegenerative diseases.^30^, ^31^, ^32^ However, there were no previous reports on the involvement of pyroptosis in the ischemic stroke. Here, we investigated the involvement of pyroptotic cell death in the pathology of cerebral ischemia provoked using a murine photothrombotic (PT) stroke model. For the first time, we show a time‐dependent increased expression of pyroptosis‐associated proteins in a stroke model. Moreover, we provide the first evidence that selectively inhibiting pyroptosis with a small molecular prodrug, Vx765, can rescue infarct volume, promote motor recovery, and improve behavioral outcomes in a murine stroke model. Mechanically, this protective effect was mediated by preventing the NLR family inflammasomes from activating the pyroptosis effector protein Gasdermin D and thus averting the disruption of plasma, nuclear, and mitochondrial membranes. Besides, our results may support the use of Vx765 as an orally active drug in future clinical trials. Indeed, the safety of Vx765 is already established in an ongoing clinical trial for treatment‐resistant epilepsy (NCT01501383); given our extremely positive preclinical results, we consider that Vx765 is poised to be fast‐tracked for translational clinical studies in cerebral ischemia.

VRT‐043198, the active metabolite of Vx765, displayed potent inhibition of ICE/caspase‐1 (Ki = 0.8 nmol/L) and caspase‐4 (Ki = 0.6 nmol/L) and at least 100‐fold lower potency against other non‐ICE subfamily caspases. VRT‐043198 exhibited no evident inhibition of trypsin or cathepsin B and only weak inhibition of granzyme B (Ki = 9 μmol/L).^11^ Murine caspase‐11 (human orthologue caspase‐4) was shown to be essential for LPS‐induced IL‐1β secretion in vivo and mediated the non‐canonical inflammasome pathway of pyroptosis that was evident only in the bacteria or LPS‐challenged mice.^33^ Thus, Vx765 treatment selectively inhibits caspase‐1 enzyme activity but has no effect on apoptosis and cellular proliferation in cellular models.^11^ Drawing upon the recently gained knowledge about GSDMD, the key protein in pyroptosis,^5^, ^7^, ^24^ we focused in this study on its role in determining the balance between pyroptosis vs neuronal survival after ischemia. GSDMD belongs to the gasdermin family^34^ and is cleaved at the D276 cleavage site mainly by the inflammatory caspase‐1, which yields the p30 N‐terminus cleavage products.^3^, ^4^ This cleavage product binds to phosphatidylserine and cardiolipin and then oligomerizes to form transmembrane pores, which then result in lytic cell death,^4^, ^7^, ^35^ as well as allowing pro‐inflammatory cytokine release.^36^ The cleavage of GSDMD at the D88 site by caspase‐3 yields the p43 fragment, which supposedly causes inactivation of GSDMD.^37^ Moreover, the p43 fragment is further cleaved at the D276 site by caspase‐8 to yield the p20 products in the context of *Yersinia* infection.^22^, ^23^ Our study showed that full‐length GSDMD, as well as its p30 and p20 cleavage products, have upregulated expression in the acute phase of cerebral ischemia. The upregulated p30 expression, in particular, hints at the involvement of pyroptosis in the pathology of acute cerebral ischemia. We saw a sharp rise of GSDMD expression in neurons of the peri‐ischemic region, whereas GSDMD exhibits little or no expression in normal cortical neurons.^38^ Our ultrastructural findings further implicate neuronal pyroptosis in cerebral ischemia. The disruption of plasma and nuclear membranes had been noted previously in an EM study of cancer cells during chemotherapy and in neurons in sepsis‐associated encephalopathy.^39^, ^40^, ^41^ A similar breakdown of mitochondria was revealed in a live‐cell analysis of triggered bone marrow‐derived macrophages.^24^ In our study, we observed plasma membrane destruction manifesting in bubbles and large holes, compromised nuclear membranes with a discontinuous out‐layer and floating debris, and damaged mitochondria with broken, but roughly intact double membranes. These findings not only support our prediction that neurons undergo pyroptotic cell death after ischemia but also shed more light on the mechanism of pore formation by GSDMD protein.

As previously mentioned, the cleavage of GSDMD by inflammatory caspase‐1/4/11 leads to irreversible pyroptosis of neurons. Inflammasomes possess a multiprotein structure, which is triggered into the assembly by multiple stimuli involving pathogen‐associated molecular patterns (PAMP) and danger‐associated molecular patterns (DAMP)^26^ to activate caspase‐1 and thus mediate pyroptosis. So far, NLR and ALR are recognized as the sensors of the two main categories of inflammasomes.^42^ Further research on the NACHT domains of NLRs revealed three subfamilies, namely the NLRPs, the IPAFs, and the NODs.^43^ In this study, we focused on the NLR family, more precisely on the NLRPs (NLRP1, NLRP3, and NLRP6) and on IPAF (NLRC4). The activation of NLRP1, NLRP3 in the peri‐ischemic region at the acute phase, in parallel with the activation of caspase‐1 and inflammatory cytokines, revealed the participation of canonical inflammatory pyroptosis in the pathology of acute cerebral ischemia.

In addition to alleviating motor deficits of PT stroke, we also found that Vx765 promoted motor learning memory. This finding is partially supported by a previous study in which Vx765 exhibited a neuroprotective effect while rescuing episodic and spatial memory impairment in a murine model of AD. Moreover, its withdrawal led to the reappearance of cognitive deficits.^30^ Our TEM finding of preserved synapses and mitochondria and the IF observation of increased neurons surviving the peri‐ischemic region together provided a structural/anatomic basis for the rescue of motor learning memory by the drug. We propose that Vx765 might also represent a promising treatment for vascular dementia and would encourage translational clinical research of Vx765 as an auxiliary medication to promote rehabilitation after acute ischemic stroke.

## CONFLICT OF INTEREST

The authors have declared that no competing interest exists. All experiments were conducted in compliance with the ARRIVE guidelines.

## Supporting information

Supplementary Material

## References

[1] Powers WJ , Rabinstein AA , Ackerson T , et al. 2018 Guidelines for the early management of patients with acute ischemic stroke: a guideline for healthcare professionals from the American Heart Association/American Stroke Association. Stroke. 2018;49(3):e46‐e110. 29367334 10.1161/STR.0000000000000158

[2] Zychlinsky A , Prevost MC , Sansonetti PJ . *Shigella flexneri* induces apoptosis in infected macrophages. Nature. 1992;358(6382):167‐169. 1614548 10.1038/358167a0

[3] Shi J , Zhao Y , Wang K , et al. Cleavage of GSDMD by inflammatory caspases determines pyroptotic cell death. Nature. 2015;526(7575):660‐665. 26375003 10.1038/nature15514

[4] Ding J , Wang K , Liu W , et al. Pore‐forming activity and structural autoinhibition of the gasdermin family. Nature. 2016;535(7610):111‐116. 27281216 10.1038/nature18590

[5] He WT , Wan H , Hu L , et al. Gasdermin D is an executor of pyroptosis and required for interleukin‐1beta secretion. Cell Res. 2015;25(12):1285‐1298. 26611636 10.1038/cr.2015.139 PMC4670995

[6] Aglietti RA , Estevez A , Gupta A , et al. GsdmD p30 elicited by caspase‐11 during pyroptosis forms pores in membranes. Proc Natl Acad Sci U S A. 2016;113(28):7858‐7863. 27339137 10.1073/pnas.1607769113 PMC4948338

[7] Sborgi L , Ruhl S , Mulvihill E , et al. GSDMD membrane pore formation constitutes the mechanism of pyroptotic cell death. EMBO J. 2016;35(16):1766‐1778. 27418190 10.15252/embj.201694696 PMC5010048

[8] Fann DY , Lee SY , Manzanero S , et al. Intravenous immunoglobulin suppresses NLRP1 and NLRP3 inflammasome‐mediated neuronal death in ischemic stroke. Cell Death Dis. 2013;4:e790. 24008734 10.1038/cddis.2013.326 PMC3789184

[9] Ismael S , Zhao L , Nasoohi S , Ishrat T . Inhibition of the NLRP3‐inflammasome as a potential approach for neuroprotection after stroke. Sci Rep. 2018;8(1):5971. 29654318 10.1038/s41598-018-24350-x PMC5899150

[10] Liberale L , Diaz‐Canestro C , Bonetti NR , et al. Post‐ischaemic administration of the murine Canakinumab‐surrogate antibody improves outcome in experimental stroke. Eur Heart J. 2018;39(38):3511‐3517. 29788103 10.1093/eurheartj/ehy286

[11] Wannamaker W , Davies R , Namchuk M , et al. (S)‐1‐((S)‐2‐{[1‐(4‐amino‐3‐chloro‐phenyl)‐methanoyl]‐amino}‐3,3‐dimethyl‐butanoy l)‐pyrrolidine‐2‐carboxylic acid ((2R,3S)‐2‐ethoxy‐5‐oxo‐tetrahydro‐furan‐3‐yl)‐amide (VX‐765), an orally available selective interleukin (IL)‐converting enzyme/caspase‐1 inhibitor, exhibits potent anti‐inflammatory activities by inhibiting the release of IL‐1beta and IL‐18. J Pharmacol Exp Ther. 2007;321(2):509‐516. 17289835 10.1124/jpet.106.111344

[12] Noe FM , Polascheck N , Frigerio F , et al. Pharmacological blockade of IL‐1beta/IL‐1 receptor type 1 axis during epileptogenesis provides neuroprotection in two rat models of temporal lobe epilepsy. Neurobiol Dis. 2013;59:183‐193. 23938763 10.1016/j.nbd.2013.07.015

[13] McKenzie BA , Mamik MK , Saito LB , et al. ,Correction for McKenzie et al.,Correction for McKenzie . Proc Natl Acad Sci U S A. 2019;116(26):13145. 31209028 10.1073/pnas.1909025116 PMC6601262

[14] Li H , Zhang N , Lin HY ,, et al. Histological, cellular and behavioral assessments of stroke outcomes after photothrombosis‐induced ischemia in adult mice. BMC Neurosci. 2014;15(1):58. 24886391 10.1186/1471-2202-15-58 PMC4039545

[15] Labat‐gest V , Tomasi S . Photothrombotic ischemia: a minimally invasive and reproducible photochemical cortical lesion model for mouse stroke studies. J Vis Exp. 2013;76:e50370. 10.3791/50370 PMC3727176 23770844

[16] Liu Y , Sun Q , Chen X , et al. Linolenic acid provides multi‐cellular protective effects after photothrombotic cerebral ischemia in rats. Neurochem Res. 2014;39(9):1797‐1808. 25062759 10.1007/s11064-014-1390-3

[17] Fiebig C , Keiner S , Ebert B , et al. Mitochondrial dysfunction in astrocytes impairs the generation of reactive astrocytes and enhances neuronal cell death in the cortex upon photothrombotic lesion. Front Mol Neurosci. 2019;12:40. 30853890 10.3389/fnmol.2019.00040 PMC6395449

[18] Cui X , Chopp M , Zacharek A , Cui Y , Roberts C , Chen J . The neurorestorative benefit of GW3965 treatment of stroke in mice. Stroke. 2013;44(1):153‐161. 23204055 10.1161/STROKEAHA.112.677682 PMC3529962

[19] Sammali E , Alia C , Vegliante G , et al. Intravenous infusion of human bone marrow mesenchymal stromal cells promotes functional recovery and neuroplasticity after ischemic stroke in mice. Sci Rep. 2017;7(1):6962. 28761170 10.1038/s41598-017-07274-w PMC5537246

[20] Ma L , Hu B , Liu Y , et al. Human embryonic stem cell‐derived GABA neurons correct locomotion deficits in quinolinic acid‐lesioned mice. Cell Stem Cell. 2012;10(4):455‐464. 22424902 10.1016/j.stem.2012.01.021 PMC3322292

[21] Liu X , Zhang Z , Ruan J , et al. Inflammasome‐activated gasdermin D causes pyroptosis by forming membrane pores. Nature. 2016;535(7610):153‐158. 27383986 10.1038/nature18629 PMC5539988

[22] Sarhan J , Liu BC , Muendlein HI , et al. Caspase‐8 induces cleavage of gasdermin D to elicit pyroptosis during Yersinia infection. Proc Natl Acad Sci U S A. 2018;115(46):E10888‐E10897. 30381458 10.1073/pnas.1809548115 PMC6243247

[23] Orning P , Weng D , Starheim K , et al. Pathogen blockade of TAK1 triggers caspase‐8‐dependent cleavage of gasdermin D and cell death. Science. 2018;362(6418):1064‐1069. 30361383 10.1126/science.aau2818 PMC6522129

[24] de Vasconcelos NM , Van Opdenbosch N , Van Gorp H , Parthoens E , Lamkanfi M . Single‐cell analysis of pyroptosis dynamics reveals conserved GSDMD‐mediated subcellular events that precede plasma membrane rupture. Cell Death Differ. 2019;26(1):146‐161. 29666477 10.1038/s41418-018-0106-7 PMC6294780

[25] Franchi L , Eigenbrod T , Munoz‐Planillo R , Nunez G . The inflammasome: a caspase‐1‐activation platform that regulates immune responses and disease pathogenesis. Nat Immunol. 2009;10(3):241‐247. 19221555 10.1038/ni.1703 PMC2820724

[26] Strowig T , Henao‐Mejia J , Elinav E , Flavell R . Inflammasomes in health and disease. Nature. 2012;481(7381):278‐286. 22258606 10.1038/nature10759

[27] Dick MS , Sborgi L , Ruhl S , Hiller S , Broz P . ASC filament formation serves as a signal amplification mechanism for inflammasomes. Nat Commun. 2016;7:11929. 27329339 10.1038/ncomms11929 PMC4917984

[28] Miao EA , Rajan JV , Aderem A . Caspase‐1‐induced pyroptotic cell death. Immunol Rev. 2011;243(1):206‐214. 21884178 10.1111/j.1600-065X.2011.01044.x PMC3609431

[29] McKenzie BA , Mamik MK , Saito LB , et al. Caspase‐1 inhibition prevents glial inflammasome activation and pyroptosis in models of multiple sclerosis. Proc Natl Acad Sci U S A. 2018;115(26):E6065‐E6074. 29895691 10.1073/pnas.1722041115 PMC6042136

[30] Flores J , Noel A , Foveau B , Lynham J , Lecrux C , LeBlanc AC . Caspase‐1 inhibition alleviates cognitive impairment and neuropathology in an Alzheimer's disease mouse model. Nat Commun. 2018;9(1):3916. 30254377 10.1038/s41467-018-06449-x PMC6156230

[31] Wang W , Nguyen LT , Burlak C , et al. Caspase‐1 causes truncation and aggregation of the Parkinson's disease‐associated protein alpha‐synuclein. Proc Natl Acad Sci U S A. 2016;113(34):9587‐9592. 27482083 10.1073/pnas.1610099113 PMC5003239

[32] Bassil F , Fernagut PO , Bezard E , et al. Reducing C‐terminal truncation mitigates synucleinopathy and neurodegeneration in a transgenic model of multiple system atrophy. Proc Natl Acad Sci U S A. 2016;113(34):9593‐9598. 27482103 10.1073/pnas.1609291113 PMC5003293

[33] Kayagaki N , Warming S , Lamkanfi M , et al. Non‐canonical inflammasome activation targets caspase‐11. Nature. 2011;479:117‐121. 22002608 10.1038/nature10558

[34] Tanaka S , Mizushina Y , Kato Y , Tamura M , Shiroishi T . Functional conservation of Gsdma cluster genes specifically duplicated in the mouse genome. G3: Genes ‐ Genomes ‐ Genetics. 2013;3(10):1843‐1850. 23979942 10.1534/g3.113.007393 PMC3789809

[35] Shi J , Gao W , Shao F . Pyroptosis: gasdermin‐mediated programmed necrotic cell death. Trends Biochem Sci. 2017;42(4):245‐254. 27932073 10.1016/j.tibs.2016.10.004

[36] Evavold CL , Ruan J , Tan Y , Xia S , Wu H , Kagan JC . The pore‐forming protein gasdermin D regulates interleukin‐1 secretion from living macrophages. Immunity. 2018;48(1):35‐44.e6. 29195811 10.1016/j.immuni.2017.11.013 PMC5773350

[37] Taabazuing CY , Okondo MC , Bachovchin DA . Pyroptosis and apoptosis pathways engage in bidirectional crosstalk in monocytes and macrophages. Cell Chem Biol. 2017;24(4):507‐514.e4. 28392147 10.1016/j.chembiol.2017.03.009 PMC5467448

[38] Tsuchiya K , Nakajima S , Hosojima S , et al. Caspase‐1 initiates apoptosis in the absence of gasdermin D. Nat Commun. 2019;10(1):2091. 31064994 10.1038/s41467-019-09753-2 PMC6505044

[39] Yu J , Li S , Qi J , et al. Cleavage of GSDME by caspase‐3 determines lobaplatin‐induced pyroptosis in colon cancer cells. Cell Death Dis. 2019;10(3):193. 30804337 10.1038/s41419-019-1441-4 PMC6389936

[40] Wang Y , Gao W , Shi X , et al. Chemotherapy drugs induce pyroptosis through caspase‐3 cleavage of a gasdermin. Nature. 2017;547(7661):99‐103. 28459430 10.1038/nature22393

[41] Xu XE , Liu L , Wang YC , et al. Caspase‐1 inhibitor exerts brain‐protective effects against sepsis‐associated encephalopathy and cognitive impairments in a mouse model of sepsis. Brain Behav Immun. 2019;80:859‐870. 31145977 10.1016/j.bbi.2019.05.038

[42] Atianand MK , Rathinam VA , Fitzgerald KA . SnapShot: inflammasomes. Cell. 2013;153(1):272‐272.e1. 23540703 10.1016/j.cell.2013.03.009

[43] Schroder K , Tschopp J . The inflammasomes. Cell. 2010;140(6):821‐832. 20303873 10.1016/j.cell.2010.01.040

