# Supplementary material for: Caspase‐1 inhibition prevents neuronal death by targeting the canonical inflammasome pathway of pyroptosis in a murine model of cerebral ischemia
Source: CNS Neurosci Ther. 2020 Apr 28;26(9):925–39. doi: 10.1111/cns.13384 (PMC7415206; doi:10.1111/cns.13384)
Supplement: Supplementary file 1 — Supplementary Material [file CNS-26-925-s001.pdf]

Figure S1 The peri ischemic region.

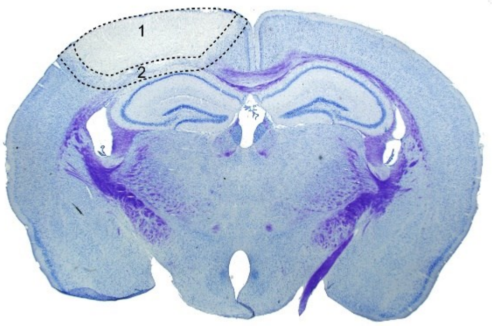

Representative Nissl stained image showed the cortical lesion, the dotted area 1 being the infarct core and the dotted area 2 is regarded as the peri-ischemic region.

Table S1 Details of reagents used in this study.

Name	Company	City	Country
Rose Bengal	Sigma Aldrich	St. Louis, Missouri	USA
Vx765	MedChemExpress	Monmouth Junction, NJ	USA
DMSO	Sigma Aldrich	St. Louis, MO	USA
anti-fade medium	Beyotime	Shanghai	China
GSDMD	Santa Cruz Biotechnology	Dallas, Texas	USA
cleaved GSDMD	Cell Signaling Technology	Danvers, MA	USA
NeuN	Cell Signaling Technology	Danvers, MA	USA
Iba1	Abcam	Cambridge	UK
GFAP	Cell Signaling Technology	Danvers, MA	USA
NG2	Abcam	Cambridge	UK
NLRP1	Santa Cruz Biotechnology	Dallas, Texas	USA
NLRP3	Adipogen	San Diego, CA	USA
caspase-1	Santa Cruz Biotechnology	Dallas, Texas	USA
ASC	Santa Cruz Biotechnology	Dallas, Texas	USA
ASC	Cell Signaling Technology	Danvers, MA	USA
NLRC4	Abclonal	Woburn, MA	USA
NLRP6	Abclonal	Woburn, MA	USA
IL-1β	Santa Cruz Biotechnology	Dallas, Texas	USA
IL-1β	Abcam	Cambridge,	UK
HRP-conjugated β-actin	Proteintech	Rosemont, IL	USA
Alexa Fluor@488, conjugated antibodies	Jackson Immuno-Research	Pennsylvania	USA
Cy3 conjugated antibodies	Jackson Immuno-Research	Pennsylvania	USA
HRP-conjugated antibodies	Jackson Immuno-Research	Pennsylvania	USA
ELISA kits	Cusabio	Wuhan, Hubei	China

Figure S2 The effects of Vx765 on the expression of NLRP1, NLRP3, ASC, CASP1 p20 and IL-1β after ischemic injuries.

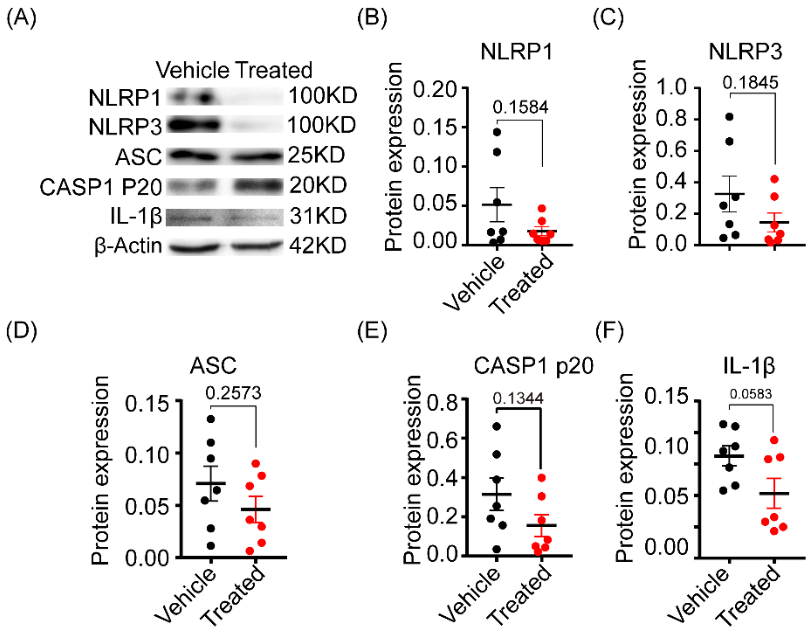

A. WB images of NLRP1, NLRP3, ASC, CASP1 p20 and IL-1β between the vehicle and treated group.

B-F. WB analyses of NLRP1, NLRP3, ASC, CASP1 p20 and IL-1β, n= seven mice per group.

Figure S3 The full unedited blots for Figure 1.

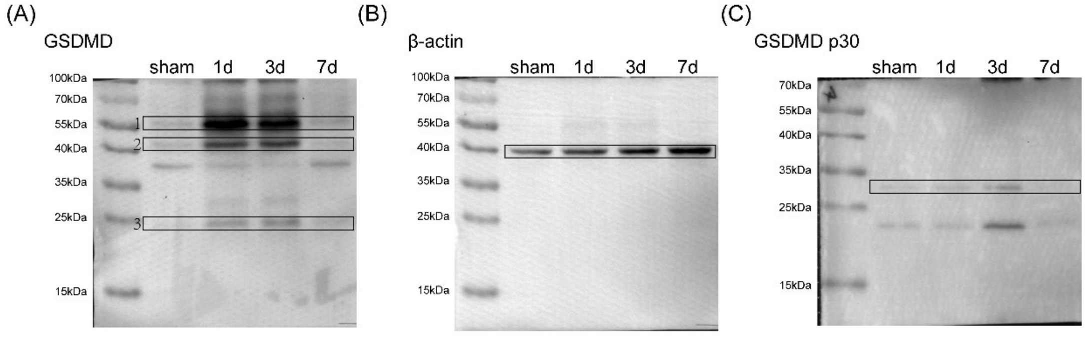

(A) The unedited blot demonstrated the full-length Gasdermin D(GSDMD), GSDMD p43 and GSDMD p20 cleavage products, lane 1 was full-length GSDMD, lane 2 was GSDMD p43, and 3 was GSDMD p20 in Figure 1H.

(B) The membrane of (A) further incubated with the β-actin antibody. The lane was β-actin in Figure 1H.

(C) The unedited blot displayed the GSDMD p30 cleavage products, the lane was GSDMD p30 products in Figure 1H.

Figure S4 The full unedited blots for Figure 3.

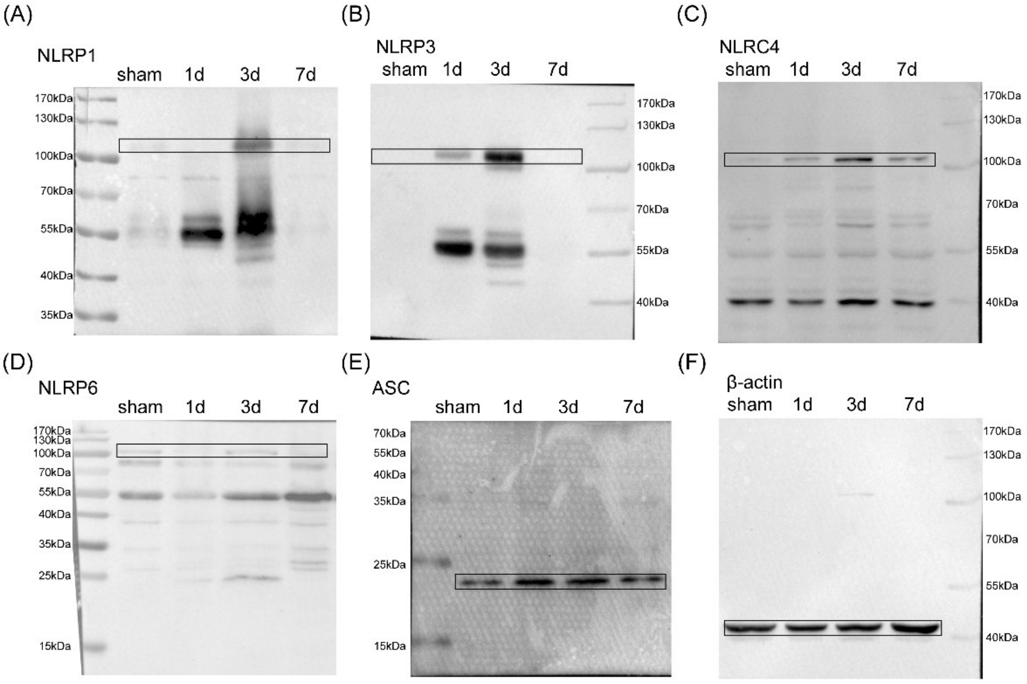

(A) The unedited blot of NLRP1, the lane was NLRP1 in Figure 3A.
(B) The unedited blot of NLRP3, the lane was NLRP3 in Figure 3A
(C) The unedited blot of NLRC4, the lane was NLRC4 in Figure 3A
(D) The unedited blot of NLRP6, the lane was NLRP6 in Figure 3A
(E) The unedited blot of ASC, the lane was ASC in Figure 3A.
(F) The membrane of (C) further incubated with the β-actin antibody. The lane was β-actin.

Figure S5 The full unedited blots for Figure 4.

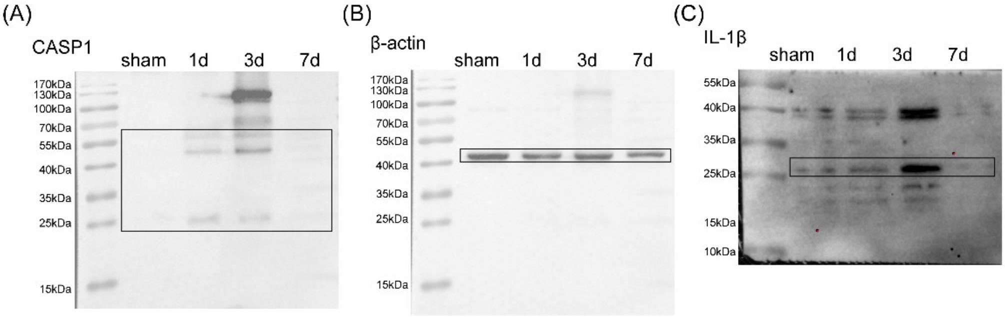

(A) The unedited blot of CASP1, the lane was caspase-1 in Figure 4G.

(B) The membrane of (A) further incubated with the β-actin antibody. The lane was β-actin in Figure 4G.

(C) The unedited blot of IL-1β, the lane was IL-1β in Figure 4G.

Figure S6 The full unedited blots for Figure 7 and Figure S2.

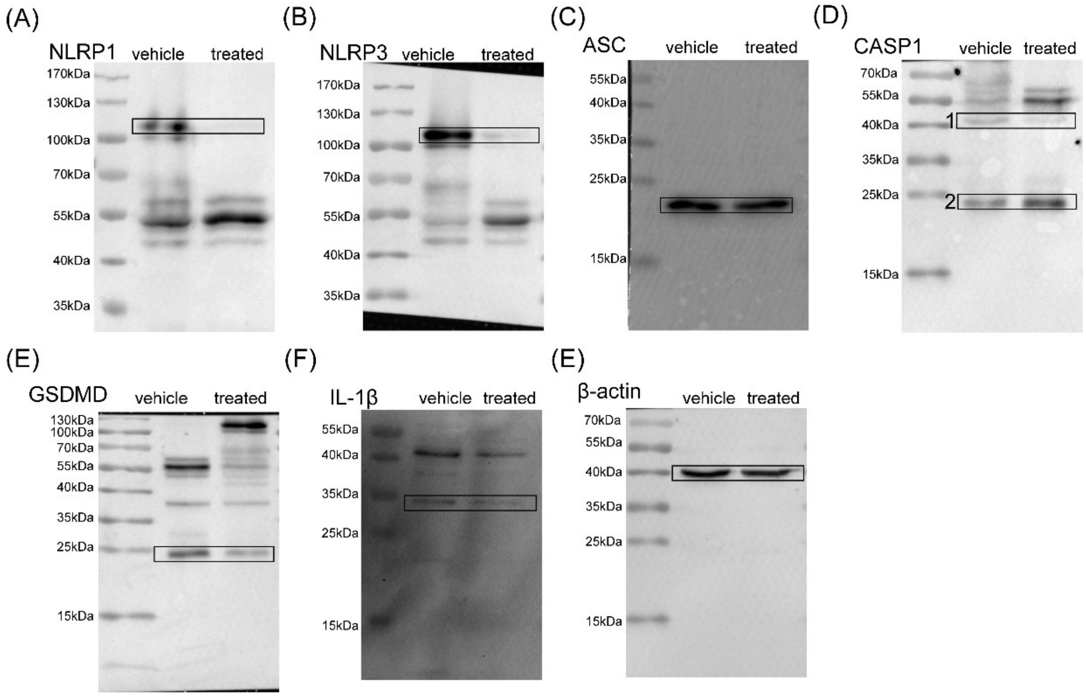

(A) The unedited blot of NLRP1, the lane was NLRP1 in Figure S2A.

(B) The unedited blot of NLRP3, the lane was NLRP3 in Figure S2A.

(C) The unedited blot of ASC, the lane was ASC in Figure S2A.

(D) The unedited blot of caspase-1, lane 1 was caspase-1 in Figure 7K and lane 2 was caspase-1 p20 in Figure S2.

(E) The unedited blot of GSDMD, the lane was GSDMD p20 in Figure 7K.

(F) The unedited blot of IL-1β, the lane was IL-1β in Figure S2A.

(E) The membrane of (D) further incubated with the β-actin antibody. The lane was β-actin in Figure 7K and Figure S2A.
